# Insights from the neural guidance factor Netrin-1 into neurodegeneration and other diseases

**DOI:** 10.3389/fnmol.2024.1379726

**Published:** 2024-04-04

**Authors:** Minqi Cai, Qian Zheng, Yiqiang Chen, Siyuan Liu, Huimin Zhu, Bing Bai

**Affiliations:** ^1^Department of Laboratory Medicine, Nanjing Drum Tower Hospital Clinical College of Jiangsu University, Nanjing, Jiangsu, China; ^2^Health Management Center, Nanjing Drum Tower Hospital, Affiliated Hospital of Medical School, Nanjing University, Nanjing, Jiangsu, China; ^3^Center for Precision Medicine, Nanjing Drum Tower Hospital, Affiliated Hospital of Medical School, Nanjing University, Nanjing, Jiangsu, China; ^4^Chemistry and Biomedicine Innovation Center, Medical School of Nanjing University, Nanjing, China

**Keywords:** Netrin-1, neurodegeneration, Alzheimer’s disease, Parkinson’s disease, psychiatric disorder, mirror movement

## Abstract

Netrin-1 was initially discovered as a neuronal growth cue for axonal guidance, and its functions have later been identified in inflammation, tumorigenesis, neurodegeneration, and other disorders. We have recently found its alterations in the brains with Alzheimer’s disease, which might provide important clues to the mechanisms of some unique pathologies. To provide better understanding of this promising molecule, we here summarize research progresses in genetics, pathology, biochemistry, cell biology and other studies of Netrin-1 about its mechanistic roles and biomarker potentials with an emphasis on clinical neurodegenerative disorders in order to expand understanding of this promising molecular player in human diseases.

## 1 Introduction

Netrin-1 is a canonical chemotropic cue for axon guidance. The discovery of netrins can be traced back to the 1890s when Dr. Cajal proposed that axons may be guided by diffusible cues that attracted the projections of spinal commissural neuron axons toward the ventral midline of the embryonic spinal cord where these cues were secreted and formed a chemotropic gradient in the neuroepithelium (Moore et al., 2007). Netrin-1, along with Netrin-2, was initially discovered and purified in embryonic chicken brain homogenate. Subsequently, other netrin family proteins have been identified or implicated in Drosophila, mice, and humans (Moore et al., 2007). Now netrins are found to not only function in axon pathfinding but also play key roles in other diverse cellular processes including, cell migration, adhesion, differentiation, and survival, with involvements in neurodegeneration (Jasmin et al., 2021), inflammation (Xia et al., 2022), cancer and other clinical diseases (Lengrand et al., 2023). Netrin-1 has been studied in Parkinson’s Disease (PD), Alzheimer’s disease (AD) and other types of neurological disorders, and we have found new evidence of Netrin-1 involved in AD pathogenesis (Bai et al., 2020). Here we provide an overview of Netrin-1 to highlight its mechanistic roles and biomarker potentials in these neurological disorders.

The netrin family proteins belong to the superfamily of laminin-like proteins and contains Netrin-1, Netrin-3, Netrin-4, Netrin-G1, and Netrin-G2 in mammals. Netrin-3 is more similar to Netrin-1 share more about 50% amino acid identity while Netrin-4 and Netrin-5 are more distinct (Rajasekharan and Kennedy, 2009). Netrin-1, Netrin-3, Netrin-4, and Netrin-5 are secreted proteins and are involved in axonal migration and neuronal growth during development of the central nervous system. In contrast, Netrin-G1 and Netrin-G2 are largely different from Netrin-1 in protein sequence and they are GPI membrane linked (Sun et al., 2011). These two proteins have not been implicated in axon guidance or neuronal growth, but have a well described role in regulating synapse formation (Rajasekharan and Kennedy, 2009; Matsukawa et al., 2014). For these distinct properties, Netrin-G1 and Netrin-G2 make up a distinct subfamily (Sun et al., 2011).

Netrin-1 is widely expressed in normal adult tissues with highest levels in the gastrointestinal tract and the muscle tissues according to the human protein atlas database^1^. In the human adult brain, it is present almost universally in all regions with a relatively high level in the midbrain. In the prefrontal cortex, it is slightly more abundant in posterior cingulate, piriform and retrosplenial cortices. However, the human tissue proteome analysis demonstrates its protein expression is selectively high at the gallbladder and the urinary bladder (Kim et al., 2014). This analysis also shows the Netrin-1 protein is highly expressed in NK cells in the peripheral blood. Other studies report its expression in macrophages, endothelium and epithelium cells (van Gils et al., 2012, 2013; Ramkhelawon et al., 2014; Bruikman et al., 2020a).

As recorded in the UniProt database^2^, Human Netrin-1 is a secreted protein and consists 604 amino acids with extensive disulfide bonds. The Netrin-1 protein contains a highly conserved a N-terminal laminin domain (typically referred to as domain VI), three cysteine-rich EGF-like repeats (referred to as domain V) and a positively charged netrin-like (NTR) module at the C-terminus with a motif of the cell attachment site (Rajasekharan and Kennedy, 2009).

Netrin-1 regulates neuronal axon guidance in human mainly through the DCC (deleted in colorectal cancer) and UNC-5 receptors (uncoordinated-5 homolog family members, UNC5A, UNC5B, UNC5C and UNC5D), and other possible receptors that include neogenin (Moore et al., 2007), DSCAM (Down syndrome cell adhesion molecule) (Ly et al., 2008; Liu et al., 2009), Adora2b (adenosine receptor A2b)(Corset et al., 2000), CD146 and integrin subunits (Stanco et al., 2009; Lemons et al., 2013; Tu et al., 2015; Li et al., 2023). The UNC5C proteins are mainly involved in axonal repulsion while the DCC receptors regulate axonal attraction through their bindings with Netrin-1 at different affinities (Boyer and Gupton, 2018). Besides, DCC and the UNC5 proteins also regulate apoptosis, either promoting it in the absence of Netrin-1 or inhibiting it in the presence of Netrin-1, and thus they are called “dependence receptors” (Llambi et al., 2001; Arakawa, 2004; Tang et al., 2008). However, these effects are also contradicted by findings in which Ntn1 null mice fails to fully recapitulate the phenotypes of Unc5−/− mice and Ntn1−/− mouse embryos exhibit increased expression of DCC and neogenin with no increased apoptosis (Bin et al., 2015; Yung et al., 2015).

The reports about DSCAM and Adora2b as netrin receptors are controversial. Subsequent to the initial description, compelling evidence has shown DSCAM to be irrelevant for netrin dependent commissural axon guidance in the embryonic spinal cord and instead appears to function as a homophilic adhesion protein that promotes axon fasciculation independent of netrins (Palmesino et al., 2012; Cohen et al., 2017). Although Netrin-1 is reported to interact with Adora2b to mediate axon outgrow and cAMP production, there is also contradictory evidence showing that Netrin-1 does not increase the concentration of intracellular cAMP in neurons (Moore and Kennedy, 2006; Moore et al., 2008).

Netrin-1 regulates axon guidance through different receptors it binds (e.g., DCC/DCC for chemoattraction, UNC5/DCC for long-range repulsion and UNC5/DSCAM for short-range repulsion), intracellular secondary messengers (e.g., cAMP, Ca++, cGMP), its own local level (low level of Netrin-1 activates DCC/DCC homodimerization to exert chemoattraction and high level of Netrin-1 induces UNC5/DCC heterodimerization for repulsion), and existence of other modulators in the extracellular environment (e.g., draxin, glycosaminoglycans, the binders of Netrin-1) (Sun et al., 2011; Boyer and Gupton, 2018). In canonical chemoattraction, Netrin-1 binds to DCC receptors and thus induces their homodimerization. This activates constitutively bound NCK1 (non-catalytic region of tyrosine kinase adaptor protein 1) and FAK (focal adhesion kinase) which starts recruitment of numerous intracellular signaling components to activate Src family kinases and Rho GTPases, release Ca++ stores, stimulate protein translation, leading to rearrangement of the actin cytoskeleton eventually (Sun et al., 2011).

Although Netrin-1 and other members in the family are extensively studied as essential chemotropic cues for migrating cells and axons during neural development, it is now evident that the netrin proteins and their receptors are also involved in other biological processes both throughout development and in adulthood, including adult stem cell migration, tumorigenesis, inflammation (Arakawa, 2004; Petit et al., 2007; Sun et al., 2011; Zhang et al., 2018; Xia et al., 2022; Cassier et al., 2023; Lengrand et al., 2023).

We recently find that the Netrin-1 expression is significantly increased in the brain tissue of patients with Alzheimer’s disease (AD), and highly correlated with Aβ in their levels. Netrin-1 colocalizes with Aβ within the plaques in both human and mouse brain tissue and starts expressing inside neurons with Aβ at the early stage. Importantly, a receptor of Netrin-1, UNC5C, has a mutation in a familial AD (Wetzel-Smith et al., 2014). These suggest that Netrin-1 is an important player in AD pathogenesis and might provide plausible explanations for some intriguing common pathologies. In consideration that Netrin-1 is also a critical factor in Parkinson’s disease (PD) and that mutation of Netrin-1 can cause neurological disorders directly, we here summarize the genetics, pathology, biochemistry, cell biology and other evidence in research progress about Netrin-1 to provide better understanding of Netrin-1 as a promising molecular in molecular mechanism and biomarker potential in these neurodegenerative diseases.

## 2 Genetic diseases of Netrin-1

The clinical significance of a molecule is best implicated by its related genetic variation and the resulting diseases. Mutations of the gene (NTN1) that encodes for Netrin-1 is associated with congenital mirror movements. This is an autosomal dominant disorder characterized by involuntary movements on one side of the body that accompany and mirror intentional movements on the other side (Online Mendelian Inheritance in Man, OMIM, # 618264). Three mutations (I518del, C601R, C601S) have been found on NTN1 that cause this type of neurological abnormality (Meneret et al., 2017). These patients have more ipsilateral corticospinal tract projections and in cultured cells proteins bearing these mutations fail to be secreted (Meneret et al., 2017). It is notable that causative gene mutations in congenital mirror movements also include DCC and RAD51, in which DCC is the receptor of Netrin-1 and RAD51 negatively regulates Netrin-1 signaling (Meneret et al., 2014; Franz et al., 2015; Glendining et al., 2017). This strengthens the potential involvement of the Netrin-1 regulatory pathway in this disease.

According to the human gene mutation database (Stenson et al., 2020), other genetic variations with NTN1 are also indicated in autism spectrum disorder (A449D) (Iossifov et al., 2014), adult-onset hearing loss (T375P) (Lewis et al., 2018), intellectual disability (V429M) or hypogonadotropic hypogonadism (R362C, T525R) respectively (Bouilly et al., 2018; Hu et al., 2019), although NTN1 are not the primary mutated gene in these diseases and further validations are needed.

## 3 Involvement of Netrin-1 in Alzheimer’s disease

Alzheimer’s disease (AD) is an aging related irreversibly progressive neurodegenerative disorder that represents about 65% of dementia cases in people over 65 years old (Tahami Monfared et al., 2022). Although some drugs such as Lecanemab have been developed and approved by FDA in America, more evidence is needed to establish their effectiveness and safeness (Burke et al., 2023; Couzin-Frankel, 2023; van Dyck et al., 2023).

AD is hallmarked by extracellular amyloid plaques and intracellular neurofibrillary tangles (NFTs) in brain cortical tissues with other frequent concomitant but not unique pathologies like amyloid angiopathy, brain atrophy, synaptic loss, white matter rarefaction, granulovacuolar degeneration, neuronal death, TDP-43 proteinopathy, neuroinflammation (Masters et al., 2015). Aβ amyloid pathology initially occurs as a few patches in poorly myelinated areas in basal parts of the neocortex, and then gradually increases and eventually spread to the entire cortex and subjacent portions of the underlying white matter (Braak and Braak, 1996). In contrast, the NFTs start early in the transentorhinal region of the medial temporal lobe and this can happen even at young ages. Later these tangles progress more severely into both entorhinal and transentorhinal regions until its culmination in neocortical and primary sensory areas eventually (Braak and Braak, 1996). It takes about 50 years from the first appearance of transentorhinal NFTs to the end stage of AD (Ohm et al., 1995), which is partially consistent with the pattern of memory and cognitive decline. Therefore, developments of Aβ plaques and NFTs do not follow the same temporal or anatomic pattern during aging in the brain. It is also notable that NFTs in the medial temporal lobe are universally present in subjects older than 70 years while Aβ plaques are found only in a significant proportion of the older population but are not universal (Nelson et al., 2012). Besides, the Aβ plaque burden in the brain is not correlated with the dementia severity while the number of NFTs are highly correlated with the number of dying neurons as well as the dementia scores (Nelson et al., 2012).

The cores of these aggregated protein deposits are Aβ peptides and hyperphosphorylated protein Tau (MAPT, microtubule-associated protein Tau) respectively (Duyckaerts et al., 2009). The Aβ peptides (40, 42, 43 and other number of amino acids long) are generated from the protein APP through sequential cleavage by the β-secretase (such as BACE1) and the γ-secretase that includes PSEN and other components probably in late endosomes and trans-Golgi apparatuses (Greenfield et al., 1999). Aβ42 is more prone to aggregation and soluble forms of these Aβ species (dimers, tetramers, oligomers) are considered more neurotoxic (Haass and Selkoe, 2007). The protein Tau has 44 phosphorylated sites and 28 of which are elevated in AD, which involves nearly 20 kinases including GSK3β (Glycogen synthase kinase-3 β), CDK5 (cyclin-dependent protein kinase-5), MAPK (mitogen-activated protein kinases), etc. (Martin et al., 2013; Tan et al., 2015; Bai et al., 2020).

So far, all three familial AD genes (APP, PSEN1, PSEN2) are directly involved in Aβ generation (Yu et al., 2021). Mutations that cause overproduction of Aβ promotes the development of AD while those that inhibit Aβ production protects people from AD (Jonsson et al., 2012). In combination with evidence from pathology, biochemistry, cell biology and animal work, it is generally posited in the field that Aβ is the initiator of this devastating disease and Tau mediates its full development (Bloom, 2014).

Besides Aβ and Tau, inflammation is another important player in AD pathogenesis (Heppner et al., 2015). Among the list of AD genetics, half of the risk factors are related to inflammation processes (Yu et al., 2021). Inflammation is considered as a central mechanism in AD (Kinney et al., 2018). First, in AD brain cortices, complement system protein components C1q, C3b, C3c, C3d, and C4 are found to localize within the amyloid plaques (Eikelenboom and Stam, 1982), and fibrillar Aβ peptides and neurofibrillary tangles can activate directly the complements *in vitro* (Tenner, 2020). AD risk genes such as TREM2, CR1, CD33, CLU are close regulators of the complement system. Besides the complements, microglia are another very important inflammatory factor in AD. Activated microglia almost universally colocalize with Aβ plaques in the AD brain tissue and they correlate with both neuropathological stages of disease severity and clinical severities of dementia (Leng and Edison, 2021). The progression and expansion of activated microglia closely parallel that of neuritic plaques in AD brains across different and regions from the hippocampus to the temporal lobe until the frontal and occipital lobes where they coexist in same cortical layers (Mrak, 2012). In addition, increased levels of pro-inflammatory mediators, such as tumor necrosis factor (TNF), IL-1β, IL-6, prostaglandins, reactive oxygen species and reactive nitrogen species, are found in brain tissues, consistent with the activation of microglia (Mrak and Griffin, 2005; Gyengesi and Munch, 2020). In fact, epidemiological and observational studies have reported that long-term treatments of inflammation diseases (such as rheumatoid arthritis) with nonsteroidal anti-inflammatory drug (NSAID) showed about 50% reduction in the risk for developing AD (Kinney et al., 2018). Inflammation in AD appears to exert a dual function, probably be neuroprotective at the early stage while lose control and become detrimental (Leng and Edison, 2021).

The involvement of Netrins in AD pathogenesis is highlighted by discovery of mutations in its receptor UNC5C in familial AD patients. A rare mutation T835M in the coding region of UNC5C segregate with AD in two families in an autosomal dominant pattern and it was associated with disease across four large case–control cohorts with the odds ratio of 2.15 (Wetzel-Smith et al., 2014). T835M is a conserved site in the hinge region of UNC5C and this mutation enhances cell death and potentiates the neurotoxicity of Aβ *in vitro* (Wetzel-Smith et al., 2014). Besides, UNC5C cleavage by δ-secretase at amino acids N467 and N547 enhances subsequent caspase-3 activation to potentiate its proapoptotic activity, facilitating neurodegeneration in AD (Chen et al., 2021). In combination with the report that UNC5H acts as a dependence receptor to induce apoptosis when the netrin ligands are absent (Llambi et al., 2001), UNC5C in the presence of Netrin-1 in AD might be considered protective. It is also notable that Netrin-5 is among the 38 genomic risk loci identified from 90,338 (46,613 proxy) cases and 1,036,225 (318,246 proxy) controls, indicating a strong association with AD as an outstanding disease mechanistic clue.

Indeed, Netrin-1 interacts with APP and modulates Aβ production and function to exert protective effects on cells and neurons. Netrin-1 is not only coimmunoprecipitated with APP from cultured cells, recruited to the plasma membrane of APP-expressing cells, but also colocalizes with APP in the growth cones of cortical neurons (Lourenco et al., 2009). This binding is mediated by involvement of several domains of Netrin-1 and the Aβ region of APP. Structural analysis reveals that Netrin-1 binds to the amino acids 4–16 of Aβ, repeatedly positioning the hydrophobic F352 side chain, toward grove 4–8 amino acids of Aβ and this is favored by the hydrophobic F4 of Aβ (Borel et al., 2017). The binding between Netrin-1 and APP leads to increased translocation of the fragment of APP intracellular domain (AICD) from cytoplasm to the nucleus, thus promoting AICD-dependent gene transcription (Lourenco et al., 2009). Mutually, through this binding, APP also regulates Netrin-1 in commissural axon navigation through the DCC receptor complex. Inactivation of APP in mice is associated with reduced commissural axon outgrowth (Rama et al., 2012).

Binding with Netrin-1 also modulates Aβ production and aggregation. It is well known that under the physiological condition, APP undergoes nontoxic cleavage by the α-secretase ADAM10 (a disintegrin and metalloprotease), while in the pathological situation of AD, β-secretase mediated cleavage to generate Aβ peptides is increased. It is found that Aβ peptides are substrates of ADAM10 and inhibit this protease, shifting the α-cleavage to β-cleavage, exerting a self-amplification effect (Spilman et al., 2016). However, this effect is inhibited by Netrin-1 in cultured cells and neurons, and Netrin-1 expression suppressed both Aβ40 and Aβ42 levels in the transgenic mice that overexpress Aβ40, probably due to binding of Netrin-1 in the Aβ region of APP, prevent it from β-cleavage to generate Aβ (Spilman et al., 2016). This provides a potential therapeutic approach for control of Aβ generation in in AD brains.

In mice, Netrin-1 restores memory performance impaired by exogenously administered Aβ. Repeated intracerebroventricular injection of Netrin-1 rescued long-term potentiation reduction and memory impairment in the maintenance phase in all cognitive behavioral tasks (Shabani et al., 2017). This might be supported by the fact that selective homozygous deletion of Netrin-1 or its receptor DCC from glutamatergic neurons in the forebrain, including hippocampal CA1 pyramidal neurons, results in significant impairment of memory consolidation (Glasgow et al., 2021), suggesting the critical role of Netrin-1 in maintenance of synaptic plasticity and thus memory in turn.

The neuronal protection from Netrin-1 on the Aβ insults might be related to its suppression on inflammation, oxidation and apoptosis. Treatment of SH-SY5Y cells exposed to Aβ42 peptides with Netrin-1 increased cell viability and partially restored the expression levels of the inflammatory factors TNFα and NF-κB and the oxidation marker nuclear factor erythroid 2–like 2 (Nrf2) (Zamani et al., 2020). The Netrin-1 treatment is also able to reduce caspase-3/7activities induced by intrahippocampal injection of Aβ42 in mice (Zamani et al., 2019). Specific mechanisms of these neuronal protective effects of Netrin-1 still remain to be clarified.

In our ultradeep mass spectrometry based quantitative proteomics analysis, we have found Netrin-1 is extremely correlated with Aβ in their levels not only in brains of human AD patients but also in those of mice that overexpress Aβ (Bai et al., 2020). Besides, Netrin-1 colocalizes with Aβ plaques in both AD human and mouse brain cortices, and it can directly bind Aβ peptides *in vitro* (Bai et al., 2020). Further experiments in our preliminary studies show that Netrin-1 starts to occur in neurons when Aβ becomes observable in 5xFAD mice at ages of 1, 2 and 3 months. From these lines of evidence, we might speculate that Aβ can possibly induce protein expression of Netrin-1 which in turn promotes Aβ aggregation through direct binding, forming a vicious cycle.

In AD, there is a prominent pathology in brain cortex that amyloid plaques are commonly surrounded by microglia and this might be related to Netrin-1. It is reported that Netrin-1 can arrest macrophages and inactivate their egress from atherosclerotic plaques (van Gils et al., 2012). Netrin-1 does so by binding to its receptor UNC5b to inhibit activation of the actin-remodeling GTPase Rac1 and actin polymerization, making macrophages anergic (van Gils et al., 2012). The same phenomenon can be seen in obesity where Netrin-1 induced by the saturated fatty acid palmitate acts through its UNC5b to retain macrophages in the adipose tissues (Ramkhelawon et al., 2014). Therefore, as the type of the macrophages in the central nervous system, microglia might also similarly be sequestered around the plaques where Netrin-1 is enriched. If this is finally turned out to be true, Netrin-1, again, should be a promising target for early therapeutic intervention of inflammation control in AD.

It is important to mention that the high correlation of Netrin-1 with Aβ is also seen in the Aβ-overexpressing 5xFAD mice, suggesting that Aβ can induce elevated protein level of Netrin-1. Because the RNA-seq data of these mice do not show altered mRNA level of Netrin-1 (Chen P. C. et al., 2022), the regulation is highly likely achieved at the protein level and this can be explained by two possible mechanisms, sequestering Netrin-1 either by direct binding or through binding of proteins that regulate Netrin-1 traffic in the secretory pathway (Kanekiyo et al., 2013; Sollvander et al., 2016; Marshall et al., 2020). If these are evidenced, the elevated Netrin-1 and other proteins (such as Midkine, Netrin-3, CTHRC, etc.) not only cause gain-of-function-like problems where they are accumulated, but also more importantly cause loss-of-function-like issues resulting from insufficient cellular secretion due to sequestration.

## 4 Involvement of Netrin-1 in Parkinson’s disease

Parkinson’s disease (PD) is the second major neurodegenerative disorder that mainly affects movement in senior population, manifesting rigidity, slowness, and tremor and other non-motor symptoms (Emamzadeh and Surguchov, 2018). It is caused by neuronal loss in the substantia nigra, resulting in insufficient synthesis of dopamine to maintain normal neuronal activities for movement control. In the brain, PD is hallmarked by intracellular inclusions of aggregated protein α-synuclein known as Lewy Bodies (Poewe et al., 2017). The underlying molecular mechanisms of pathogenesis include α-synuclein proteostasis, mitochondrial function, oxidative stress, calcium homeostasis, axonal transport, neuroinflammation and other potential biological processes and cellular signaling (Poewe et al., 2017).

Netrin-1 and its receptor DCC are highly expressed in adult brains in dopaminergic neurons of the substantia nigra pars compacta (SNpc) which is selectively affected in PD (Osborne et al., 2005). Netrin-1 can also be produced in the forebrain and transferred through axons to the midbrain, to direct migration of GABAergic neurons into the ventral SN during development, confining dopaminergic (DA) neurons within the dorsal SN (Brignani et al., 2020). Netrin-1 acts on DA neurons at both ventral tegmental area (VTA) and SN, but these two populations of DA axons respond differentially: VTA axons prefer higher concentrations while SN axons require lower concentrations, so that topographic distribution of specific neuron types can be maintained (Li et al., 2014).

According to recent data, Netrin-1 may be associated with PD pathogenesis. During aging, Netrin-1 is substantially reduced in the brain and this is more significant in PD patient brains although this is possibly due to loss of dopaminergic neurons which are the major source of Netrin-1 (Ahn et al., 2020b). In PD mouse models, imbalance of NTN-1 and DCC is found to be a common feature in nigral DA neurons in which the well-established chemical PD inducer MPP+ (1-Methyl-4-phenyl pyridinium iodide) inhibits the expression of Netrin-1 but increases DCC expression in both concentration- and time-dependent manners (Hua et al., 2023). Normally, only Netrin-1 is significantly expressed in the substantia nigra of healthy adult brains while α-synuclein is basally present and their protein levels are inversely correlated. It is actually found that Netrin-1 and α-synuclein can directly interact with each other and Netrin-1 blocks α-synuclein aggregation *in vitro*; besides, Netrin-1 deprivation initiates α-synuclein aggregation in cultured primary DA neurons (Kang et al., 2023). Therefore, loss of Netrin-1 enhances α-synuclein aggregation and possibly contributes to PD pathogenesis. *In vivo*, conditional knockout of Netrin-1 specifically in the adult mouse induces DCC cleavage and a significant loss of dopamine neurons, leading to impaired motor function in these mice (Jasmin et al., 2021).

Chronic constipation is a frequent symptom that occurs even before the onset of PD and propagation of the aggregated α-Synuclein-containing Lewy bodies from the gut into the brain has been proposed as a key mechanism in PD etiopathogenesis (Braak et al., 2006). PD mice demonstrate increased intestinal permeability to proinflammatory bacterial products (Kelly et al., 2014), imposing the oxidative stress on the enteric neurons (Forsyth et al., 2011). Research has shown an inverse correlation of Netrin-1 and BDNF (brain-derived neurotrophic factor) with inflammatory cytokines-activated transcription factor CCAAT/enhancer binding protein β (C/EBPβ) in PD patient brains and colons resulting from binding of C/EBPβ to the promoters of Netrin-1 and BDNF genes to inhibit their mRNA expression (Ahn et al., 2020a, 2021).

There are two possible mechanistic pathways that have been studied in the death of DA neurons caused by Netrin-1 insufficiency: one is the mammalian Ste20-like kinases 1 (MST1) and the other is the delta-secretase (asparagine endopeptidase, AEP). The MST1/2 is involved in the Hippo pathway that is critical in controlling tissue growth, cell proliferation, differentiation, and migration in developing organs. Netrin1 reduction activates MST1 which in turn selectively binds and induces phosphorylatin of UNC5B on T428 to generate its apoptotic fragment via active caspase-3 in dopaminergic neurons in the SN. Netrin1 deprivation also causes the downregulation of YAP, a protein involved in ROS scavenge. Both pathways lead to dopaminergic neuronal death. Besides, deficiency of Netrin-1 activates of delta-secretase (asparagine endopeptidase, AEP) which then cleaves both α-Synuclein at N103 and the UNC5C receptor in an age-dependent manner in mice, resulting in accelerated DA neuronal loss and PD phenotypes and pathologies, which can be rescued by AEP deletion. Notably, AEP is highly active in the SNpc regions in human brains with PD where the DA neurons are mainly located and Netrin-1 is highly expressed (Wang et al., 2018; Ahn et al., 2021). However, these are challenged by findings as mentioned earlier in which Ntn1 is unlikely the dominant ligand for Unc5 family and Ntn1−/− mouse embryos exhibit increased expression of DCC and neogenin but no increased apoptosis (Bin et al., 2015; Yung et al., 2015). Besides, deletion of DCC, which is proposed to be pro-apoptotic by the dependence model, is instead required for dopaminergic neuronal survival during aging (Lo et al., 2022), suggesting the impact of Netrin-1 on cell survival is more complex than the mechanisms proposed by the dependence model.

Overall, Netrin-1 is critical to maintain healthy DA neurons in SN and its deficiency is probably one of the key mechanisms in PD etiology.

## 5 Netrin-1 in psychiatric disorders

Many psychiatric disorders are related to the mesocorticolimbic dopamine system where dopamine cells project from the upper brainstem to the dorsal striatum and multiple cortical and subcortical limbic regions including the ventral striatum nucleus accumbens, olfactory tubercle, septum, hippocampus, amygdala, and cortical regions, particularly the prefrontal cortex, cingulate, and the perirhinal cortex (Vosberg et al., 2020). Genetic variations of Netrin-1/DCC have been shown to associate significantly with depression, schizophrenia, and substance use (Flores, 2011; Hoops and Flores, 2017). This is further evidenced by two other Netrin family members: Netrin-G1 and Netrin-G2 which have shown significant associations in schizophrenia (Aoki-Suzuki et al., 2005). Besides, according to the largest genome-wide meta-analysis of psychiatric disorders conducted so far (~725,000 cases-controls, across eight psychiatric disorders), the intronic DCC SNP rs8084351 shows the most significant and pleiotropic effect (Cross-Disorder Group of the Psychiatric Genomics Consortium, 2019; Torres-Berrio et al., 2020). In addition, results from human postmortem examinations, animal work and GWAS studies suggest that the relevance of the Netrin-1/DCC pathway in the etiology of major depressive disorder due to its abnormal spatiotemporal organization of circuits involved in cognition and emotion (Torres-Berrio et al., 2020). In mice, Dcc haploinsuffificiency results in impaired dopamine transmission and dopamine-related behaviors in adulthood (Flores, 2011; Hoops and Flores, 2017).

## 6 Netrin-1 in other clinical diseases

The Netrin-1 has already been extensively studied in other diseases such as inflammation, angiogenesis, diabetes, atherosclerosis and tumorigenesis (Xia et al., 2022). In the acute inflammation of ischemia–reperfusion (I/R) injury, the protein level of Netrin-1 is reduced in the affected tissues such as kidney, liver, lung and myocardium (Zhang and Cai, 2010; Ranganathan et al., 2013; Schlegel et al., 2016). Mice heterozygous for Netrin-1 deficiency (Ntn1+/−) undergo more activated inflammation and manifest severer hepatic I/R injury (Schlegel et al., 2016). Treatment with Netrin-1 or its peptides in cultured cells or mice largely alleviates inflammation (Zhang and Cai, 2010; Bouhidel et al., 2014; Cui, 2015; Boneschansker et al., 2016; Liu et al., 2019; Chen et al., 2020b). Netrin-1 exerts these protections by inhibiting production of cytokines (such as IL-2, IL-4, IL-6, IL-13, IL-17, interferon-γ, etc.) and suppressing expression of cyclooxygenase-2 and prostaglandin E2 in T regulatory cells, polymorphonuclear neutrophils and macrophage (Xia et al., 2022), and thus to regulate activation, filtration and polarization of these major inflammatory cells. Specific signaling pathway for these effects involve Netrin-1 and its receptors. For examples, in cardiac I/R, the perfused Netrin-1 binds to its receptor DCC and thus activates the ERK1/2/eNOS pathway to maintain DCC expression via a feed-forward loop and promote generation of nitric oxide (NO·) to protect heart tissues from infarct apoptosis (Zhang and Cai, 2010).

In angiogenesis, Netrin-1 exerts a promoting or inhibiting effect depending on the receptors it binds to or its protein concentration. Upon binding to DCC, Netrin-1 activates the downstream ERK1/2 signaling to phosphorylate eNOS for increase production of endothelial NO, forming a feed-forward signaling cascade to promote angiogenesis (Nguyen and Cai, 2006). Netrin-1 also binds to CD146 or an unknown receptor to enhance endothelial cell growth and migration possibly through other mechanisms (Park et al., 2004; Tu et al., 2015). However, when bound to the receptor UNC5B, Netrin-1 exerts repulsive effects in angiogenesis including endothelial filopodial extension, vessel branching and abnormal navigation (Lu et al., 2004). The bidirectional effects of Netrin-1 in angiogenesis are also concentration-dependent: at low levels, Netrin-1 induces endothelial proliferation, migration and tube formation while at higher doses these effects are inhibited (Xia et al., 2022).

Netrin-1 is also an important molecular player in atherosclerosis, exerting beneficial or disastrous effects depending on its cellular source (Xia et al., 2022). Netrin-1 secreted by endothelial cells is protective as it inhibits chemotaxis of leukocytes and migration of monocytes to atherosclerotic plaques. In contrast, macrophage-derived Netrin-1 is proatherogenic in that it retains macrophage numbers in the plaques (Fiorelli et al., 2021). Besides, a mutation (R590L) within NTN1 is found in a family with premature atherosclerosis, strongly suggest the causative role of Netrin-1 in this common disease (Bruikman et al., 2020b).

In diabetes mellitus and its complications, Netrin-1 is highly expressed in obese adipose tissue of humans and mice, causing retention of macrophages for activated inflammation (Ramkhelawon et al., 2014). It is also significantly altered in the peripheral circulation system (Ay et al., 2016; Liu et al., 2016; Yim et al., 2018). It regulates pancreatic epithelial cell migration and tissue regeneration as well as β-cell apoptosis (De Breuck et al., 2003; Yang et al., 2011). Mice with partial Netrin-1 deficiency demonstrate severer kidney injury in with diabetic nephropathy which can be restored by treatment with recombinant Netrin-1 (Tak et al., 2013). In mice with high-fat diet/streptozotocin-induced diabetes, Netrin-1 treatment increases insulin release from β-cells, promotes islet vascularization, reduces islet macrophage infiltration, and alleviates inflammation (Gao et al., 2016).

The study of Netrin-1 is heated in the cancer field. Netrin-1 is often highly expressed in cancer tissues and involved in tumorigenesis as an oncogene (Arakawa, 2004; Mehlen et al., 2011; Sung et al., 2019). Normally it is expressed mainly during embryonic development, but probably due to the function of anti-apoptosis through its death receptors, Netrin-1 has been found to be highly expressed in tissues of many tumors, including inflammation-associated colorectal cancer (Paradisi et al., 2008, 2009), metastatic breast cancer (Fitamant et al., 2008), endometrial cancer (Cassier et al., 2023), lung cancer (Delloye-Bourgeois et al., 2009a), neuroblastoma (Delloye-Bourgeois et al., 2009b), lymphoma and melanoma (Broutier et al., 2016; Boussouar et al., 2020). According to the globally largest and most comprehensive cancer mutation database (Catalogue of Somatic Mutations in Cancer, COSMIC), point mutations, copy number variation, high expression or methylation of NTN1 is found in almost 30 types of cancers. In animal models, downregulation of Netrin-1 or its receptors promotes cancer cell death and inhibits tumor growth (Broutier et al., 2016; Sung et al., 2019; Boussouar et al., 2020). Based on the causative role of Netrin-1 in cancer pathogenesis and extensive studies showing its efficiency as a therapeutic target, a monoclonal antibody (NP137) that targets Netrin-1 to disrupt its interaction with the UNC5B receptor has been developed and is currently receiving clinical phase 1 trial (NCT02977195) to evaluate safety and efficacy (Grandin et al., 2016; Cassier et al., 2023). The current studies about Netrin-1 in the cancer field is moving rapid and more achievements can be expected in the near future.

## 7 Biomarker potentials of Netrin-1

Netrin-1 is a secreted protein and can thus be released from the affected tissue regions into body fluid, especially serum, becoming potential biomarkers. In AD, the serum Netrin-1 protein levels are lowered in AD and MCI (mild cognitive impairment) patients and correlated with reduction in dementia scores (Ju et al., 2021). Interestingly, a bilateral intracerebroventricular injection of Aβ42 in rats has not only induced spatial learning and memory deficits and increased neuronal apoptosis, but also reduced Netrin-1 protein levels in both serum and cerebrospinal fluid of these rats with a significant correlation with cognitive deficits (Sun et al., 2019). Besides AD, the serum Netrin-1 is also found to decrease in clinical patients with spinal cord injury and can be an independent risk factor for cognitive impairment in these patients (Meng et al., 2022).

In PD, as the substantia nigra where Netrin-1 is highly expressed is the most affected area in this disease, reduced Netrin-1 protein expression is found in these affected regions of brain tissues from PD patients (Jasmin et al., 2021). Intriguingly, the reduced Netrin-1 level is not only found in PD brain tissues, but also found in the gut where Lewy body-like aggregation first appears in the enteric neurons even before its occurrence in the brain (Ahn et al., 2021). Loss of these neurons that are the major sources of Netrin-1 in these areas might lead to reduced Netrin-1 in the peripheral system. A recent study reveals a significant decrease in plasma Netrin-1 levels with a positive correlation with UPDRS (Unified Parkinson’s Disease Rating Scale) scores in PD patients (Hua et al., 2023), strongly supporting its biomarker potential in this second most common neurodegenerative disease.

Besides the aging-related neurodegenerative disorders, the reduced serum netrin-1 level is also found in ischemic stroke and its complications (Guo et al., 2020; Chen Z. et al., 2022), post-stroke depression (Chen et al., 2020a), and delayed neurological sequelae in unintentional carbon monoxide poisoning (Kokulu et al., 2020); and increased serum Netrin-1 predicts better prognosis of ischemic stroke (Guo et al., 2019; Zang et al., 2021). In fact, altered Netrin-1 levels in serum, urine or types of body fluids are associated with a large number of other clinical diseases, including cancer (Kefeli et al., 2017), atherosclerosis (Munoz et al., 2017; Bruikman et al., 2020a), obesity and diabetes (Yim et al., 2018; Elkholy et al., 2021; Nedeva et al., 2022), kidney Injury (Reeves et al., 2008; Ramesh et al., 2010), brain damage and hemorrhage (Chen et al., 2019; Lou et al., 2020; Xie et al., 2021), periodontitis (Gunpinar et al., 2020; Abdulfattah et al., 2022), acute coronary syndrome (Leocadio et al., 2020), sclerosis (Mulero et al., 2017), preeclampsia (Cekmez et al., 2017; Berenji et al., 2022; Sert, 2022; Kaya et al., 2023).

In cancer, the expression of Netrin-1 is largely altered in lesioned tissues, but its level in serum has not been extensively studied. Increased serum Netrin-1 is found in gastric and lung cancers and it is reduced after chemotherapy, but the levels do not show correlations with the patient survivals (Kefeli et al., 2012; Yildirim et al., 2016). In a large scale of clinical blood samples from cancer patients, plasma Netrin-1 levels are significantly higher in breast, renal, prostate, liver, meningioma, pituitary adenoma, and glioblastoma cancers than it in controls (Ramesh et al., 2011). Recent studies report serum netrin-1 a novel biomarker in colorectal cancer and lung cancer (Li et al., 2020; Zhao et al., 2022).

Although the Netrin-1 level in the peripheral system is changed widely in a large number of diseases, it might bear the potential as a biomarker in diseases of the similar type (such as cancer) or sharing a similar mechanism (such as inflammation).

## 8 Conclusion

Here we have reviewed Netrin-1 by its genetics, pathology, biochemistry and other biological evidence about its mechanistic involvement and biomarker potential in neurodegenerative, inflammatory, cancerous and other diseases. Mutation of the Netrin-1 gene can cause neurological and other diseases directly, implicating it is not indispensable importance in maintaining normal physiological function. Netrin-1 is highly likely protective in AD, but is also possibly responsible for microglia attraction in the brain tissue. Netrin-1 is critical to dopaminergic neurons in substantia nigra of the brain and its deficiency is critical in both PD pathogenesis and development of psychiatric disorders. It is largely protective in inflammation and the related diseases, bidirectionally effective in atherosclerosis, and generally deleterious as an oncogene in cancer. Besides, Netrin-1 might also be a potential biomarker for these clinical diseases. Here we summarize all these in the Table 1 for better understanding of this promising molecule. Overall, Netrin-1 is a promising protein to be studied across a variety of disease spectra for the discovery of novel molecular mechanisms, potential biomarkers, and therapeutic targets.

**Table 1:** List of neurological disorders and other clinical diseases that involves Netrin-1.

**Diseases**		**Involvement of Netrin-1 and its signaling pathway**	**Netrin-1 level in clinical sample**	**Reference** (PMID)
1. Mirror movements		I518del, C601R, C601S in NTN1; S126X, N176S, W273R, R275X, G470D, R667H, N702S, V793G, G803R in DCC (the Netrin-1 receptor); H47R, I136F, R250Q, R254X, a heterozygous one base pair duplication (855dupA) in exon 9 in RAD51 (a negative Netrin-1 signaling regulator)		28945198, 24808016
2. Alzheimer’s Disease (AD)		Mutation of the Netrin-1 receptor UNC5C (T835M) is associated with familial AD patients.	Serum Netrin-1 is reduced in AD and MCI, and patients with cognitive decline due to spinal cord injury.	25419706
		Netrin-1 interacts with APP and modulates Aβ production.		19148186
		Netrin-1 inhibits apoptosis, inflammation, oxidation, and protects mice from memory loss and cognitive decline induced by Aβ.		27060954, 28389207
		Netrin-1 is highly correlated with Aβ in levels in the brain tissue of both human AD patients and the mice that overxpressed Aβ (5xFAD); colocalizes with Aβ in amyloid plaques.		31926610, 35250531, 35493300
3. Parkinson’s disease (PD)		Netrin-1 and its receptor DCC are highly expressed in adult brains in dopaminergic neurons of the substantia nigra which is affected in PD.	Serum Netrin-1 is reduced in PD.	36852451
		Netrin-1 is more significantly reduced in brains of PD patients.		32929029
		Netrin-1 binds α-synuclein and their levels are inversely correlated in substantia nigra.		36751943
		Chronic constipation occurs frequently in PD at the early stage during which the aggregated α-Synuclein-containing Lewy bodies is often found in the gut		16330147
4. Psychiatric disorders		The psychiatric disorders are related to the mesocorticolimbic dopamine system which involves the Netrin-1/DCC signaling pathway.		31659271, 32593422, 31835028, 21481303, 29032842
		Genetic variations of Netrin-1/DCC have been shown associate significantly with depression, schizophrenia, and substance use.		
		The DCC expression in the prefrontal cortex is altered in major depressive disorder and determines susceptibility to chronic social defeat stress.		
		In mice, the haploinsuffificiency of the Netrin-1 receptor Dcc causes impaired dopamine transmission and dopamine-related behaviors in adulthood.		
5. Other diseases				
	Ischemia–reperfusion (I/R) injury	Reduced in affected kidney, liver, lung and myocardium tissues.		35082104, 26573873
		Ntn1^+/−^ mice show stronger inflammation and severer hepatic I/R injury.		
		Netrin-1 treatment protects inflammation and angiogenesis by inhibiting inflammatory cells and their release of cytokines.		
		Netrin-1 exerts protective effects through DCC/ERK/eNOS pathway.		
	Atherosclerosis	Netrin-1 was downregulated and UNC5B upregulated in atherosclerotic plaques.	Netrin-1 plasma levels are lower in subclinical atherosclerosis and negatively correlated with plaque burden.	24122613, 37439909, 32151395
		Netrin-1 is generally considered to protect atherosclerosis by inhibiting inflammation, but conflicting reports also exist.		
		A NTN1 mutation (R590L) is associated with a familial premature atherosclerosis.		
	Diabetes	Netrin-1 is highly expressed in obese adipose tissue of humans and mice.	The reported blood levels of Netrin-1 is inreased or reduced, controversial.	21212933, 27520508, 37996774
		Netrin-1 treatment promotes adult β-cell survival and insulin release.		
	Cancer	Netrin-1 protein expression is increased in tissues of many types of cancer such as colorectal cancer (from inflammatory bowel diseases), metastatic breast cancer, non-small cell lung cancer, neuroblastoma, melanoma.	Plasma or serum netrin-1 levels are significantly increased in lung, breast, prostate, colorectal, renal, liver, meningioma, pituitary adenoma, and glioblastoma cancers.	15573119, 37532929, 37532934
		NP137, a netrin-1-blocking monoclonal antibody is currently in clinical trials for human cancer therapy (ClinicalTrials.gov identifier NCT02977195).		21303223, 27067437, 32474463, 35765995
	Ischemic stroke and its complications		Serum Netrin-1 level is reduced.	32417215, 32912541, 30852966
	Post-stroke depression		Serum Netrin-1 level is reduced.	32912541
	Delayed neurological sequelae in unintentional carbon monoxide poisoning		Serum Netrin-1 level is reduced.	32228196
	Brain hemorrhage		Serum Netrin-1 level is reduced.	31047878
	Sclerosis	Lower Netrin-1 protein expression in the spinal cord and the cerebella of experimental mice.	Serum Netrin-1 level is reduced.	28677863
	Preeclampsia		Serum Netrin-1 level is increased.	27296221
	Acute coronary syndrome		Serum Netrin-1 level is high.	32267322
	kidney Injury		Urinary netrin-1 level is increased.	18234954, 20007677
	Periodontitis		Netrin-1 level in the gingival crevicular fluid is increased.	31769036

## Author contributions

MC: Data curation, Investigation, Methodology, Writing – original draft, Writing – review & editing. QZ: Conceptualization, Data curation, Project administration, Writing – original draft. YC: Methodology, Resources, Validation, Writing – review & editing. SL: Methodology, Resources, Validation, Writing – review & editing. HZ: Supervision, Validation, Writing – review & editing. BB: Conceptualization, Data curation, Funding acquisition, Methodology, Supervision, Writing – review & editing.

## Footnotes

^1^ https://www.proteinatlas.org/

^2^ https://www.uniprot.org/
